# The Role Of Physical Activity On The Prevention Of Cognitive Impairment

**Published:** 2016-01-31

**Authors:** G. Corbi, V. Conti, A. Filippelli, A. Di Costanzo, N. Ferrara

**Affiliations:** 1 Dpt of Medicine and Health Sciences. University of Molise; 2 Dpt of Medicine and Surgery, University of Salerno; 3 Dpt of Translational Medical Sciences, University of Naples “Federico II” Naples

**Keywords:** sirtuins, amyloid, beta adrenergic

## Abstract

Physical exercise is associated with reduced risk of heart disease, type II diabetes mellitus, and overall mortality. However, growing evidence shows that physical activity can also improve cognitive function and may lower the risk of developing dementia, but Randomized Clinical Trials gave mixed results. Aim of this article was to review the knowledge available in literature on the effects of physical activity on cognition and the suggested possible mechanisms involved in these effects. Our group have planned a trial aiming to evaluate the effectiveness of physical activity in preventing or delaying the cognitive decline in individuals at risk of developing dementia. Beside the effects of exercise on cognition are not fully defined, also the mechanisms underlying the benefits of physical activity on cognitive sphere are not completely known. Recently the SIRT1 loss is both closely associated with accumulation of beta amyloid and tau protein in AD patients. Although there is no specific exercise that can be recommended, the available evidence suggests that practicing more types of physical activity is particularly advantageous. It is important to explore further mechanisms involved in the pathogenesis of the AD in order to be able to identify new and effective target treatment, including physical activity.

## I. INTRODUCTION

Physical exercise is associated with reduced risk of heart disease, type II diabetes mellitus, some types of cancers, and overall mortality [1] and benefits are also related to the anti-inflammatory effects on chronic degenerative diseases such as atherosclerosis, hypertension COPD, etc. [2–6]. There is growing evidence that physical activity can also improve cognitive function in the short term and may lower the risk of developing dementia over the long term. Numerous observational studies [7–16] and several systematic reviews [17,18] have found that elderly undergone to physical activity are less likely to experience cognitive decline or develop dementia, and several studies have suggested that physical activity may have independent or additive effects [7,11].

As cumulative neuronal loss is considered critical in the pathological process of the dementias [19], it has been proposed that interventions targeted at preserving neuronal function in Mild Cognitive Impairment may prevent or postpone cognitive decline into dementia [20]. However, to date Randomized Controlled Trials (RCTs) on physical activity interventions gave mixed results [21], therefore aim of this article was to review the knowledge available in literature on the effects of physical activity on cognition and the suggested possible mechanisms involved in these effects.

## II. TRIALS ON PHYSICAL ACTIVITY IN HUMANS

Exercise interventions in both healthy older adults [22, 23] and individuals with mild cognitive impairment [24–26] have found that aerobic exercise and resistance training are associated with small to moderate improvements in cognitive function, mainly regarding measures of attention, processing speed and executive function [27–29].

Verghese et al [30] reported that increased participation in leisure activities was associated with lower risk of dementia as well as reduced global cognitive decline in the Bronx Aging Study. After, the same authors showed that high levels of participation in cognitive leisure activities is associated with reduced risk of amnestic Mild Cognitive Impairment (aMCI) in community-residing older adults, initially free of MCI or dementia. A one-point increase in the cognitive activity scale score was associated with a 5% reduced risk of aMCI. The observed association remains robust even after adjusting for potential confounders such as age, sex, education, chronic illnesses, depression, and baseline cognitive status. These results suggested a dose response effect of physical activity in MCI and dementia prevention [31].

Also Barnes et al [32] showed that in inactive older adults with cognitive complaints, 12 weeks of physical plus mental activity were associated with significant improvements in global cognitive function with no evidence of difference between intervention and active control groups. The authors proposed that these findings may reflect practice effects or may suggest that the amount of activity is more important than the type in this subject population.

In contrast, Sink et al [33] recently designed a randomized clinical trial, the Lifestyle Interventions and Independence for Elders (LIFE) study. The study population was represented by 1635 community-living participants sedentary adults aged 70 to 89 years who were at risk for mobility disability but able to walk 400 m. A structured, moderate-intensity physical activity program that included walking, resistance training, and flexibility exercises or a health education program of educational workshops and upper-extremity stretching was performed. No differences for any cognitive or composite measures were observed. Then the authors concluded that, among sedentary older adults, a 24-month physical activity program compared with a health education program did not result in improvements in global or domain-specific cognitive function.

Then starting by the evidences that still conflicting data are available in literature, our group have planned a randomized controlled (the Exercise and Prevention of Dementia [EPD]) clinical trial with the aim to evaluate the effectiveness of physical activity in preventing or delaying the cognitive decline in individuals at risk of developing dementia, namely in patients with subjective memory disorder or mild cognitive impairment (MCI), following the CONSORT recommendations. In particular, subjects older than 60 years old, consecutively admitted at the Centre for the Education and Research on Medicine for Aging (CERMI), after general and neuropsychological evaluation were randomized to undergo to active exercise or stretching. The intervention group consisted of subjects that performed aerobic controlled moderate exercise training, while the control group undergo only to stretching exercises. All study population were evaluated at 6, 12, 24, 48 months. Currently the statistical analysis is being carried out.

## III. PHYSICAL ACTIVITY EFFECTS ON NEUROGENESIS

Beside the effects of exercise on cognition are not fully defined, also the mechanisms underlying the benefits of physical activity on cognitive sphere are not completely known. Some studies indicate a positive correlation between exercise and hippocampal volume front [34]. In addition, studies with functional magnetic resonance imaging show an increased connectivity between cortex frontal and temporal levels similar to those found in young individuals. One of the phenomena most often associated with physical activity is the increase of the proliferation and survival cell hippocampus [35] and, therefore, neurogenesis. Phenomena of neurogenesis starting from neonatal cells could help to explain the effects of exercise on the learning and memory functions. One of the molecules that are believed implicated in these processes is the brain-derived neurotrophic factor (BDNF) whose levels are increased by physical activity, and closely correlated with the improving learning and memory described in trained rodents [36]. Two other molecules potentially involved in the effects of exercise on cognitive functions are the vascular endothelial growth factor (VEGF) and the insulin-like growth factor 1 (IGF1), responsible for the activation of brain angiogenesis [37]. In addition, the physical activity is able to favourably control oxidants/antioxidants systems, in favour of these last [38–40], through the stimulation of lifespan molecules such as sirtuins [41,42], with a dependent effect on both the type [43], and the degree of intensity of physical activity [44].

Recently it was shown how the loss of SIRT1, the human homolog of Sir2, was closely associated with accumulation of beta amyloid and tau protein in the cerebral cortex of patients with Alzheimer’s disease (AD) [45]. In fact, although in recent decades the pathogenetic mechanisms of AD have been the subject of numerous studies, many aspects of the disease remain to be clarified and therapeutic strategies currently available are not able to modify significantly its progression, but rather induce an improvement in symptoms.

In particular, in recent years the pathogenetic “neurocentric” hypothesis of the AD, focused mainly on the neuronal damage due to an excess of beta-amyloid, has given way to a more extended “neurovascular” hypothesis. This switch was caused by the evidence that an early vascular damage contributes to the development of the AD, and the observation that many risk factors associated with the development of AD are also known cardiovascular risk factors [46].

In this context, it has been formulated the hypothesis that alterations of the adrenergic system, the important role of which on cardiovascular homeostasis is well known, may contribute to the pathogenesis of AD. In this sense, some evidences have showed that in course of AD the noradrenergic brain system undergoes several changes, and that in particular the stimulation of beta-adrenergic receptors is involved in the production of pathological amyloid in animal models [47,48]. This mechanism seems to involve also kinases associated with the adrenergic receptors, and in particular the G protein coupled receptor kinase 2 (GRK2), which plays a key role in the downregulation and desensitization of the adrenergic receptors [49–51]. Furthermore, overexpression of this kinase was found in the vascular lesions in models of but [52].

In this scenario, physical activity may play an important role in the normalization of these molecular events, given its well-known ability to interfere favourably on adrenergic signalling in the cardiovascular system [53].

Therefore, it is of fundamental importance to explore further mechanisms involved in the pathogenesis of the AD in order to be able to identify new and effective target treatment, including physical activity.

## IV. CONCLUSION

Although there is no specific exercise, which can be recommended, the available evidence suggest that practicing more types of physical activity, especially in a group, is particularly advantageous. To increase levels of physical activity in community, revitalization and other approaches, such as the establishment of local networks, including sports clubs, and appropriate interventions of public health are needed.
